# Cardiac Pacemaker Dysfunction Arising From Different Studies of Ion Channel Remodeling in the Aging Rat Heart

**DOI:** 10.3389/fphys.2020.546508

**Published:** 2020-12-03

**Authors:** Aaazh M. Alghamdi, Mark R. Boyett, Jules C. Hancox, Henggui Zhang

**Affiliations:** ^1^Biological Physics Group, Department of Physics and Astronomy, The University of Manchester, Manchester, United Kingdom; ^2^Department of Physics, Faculty of Science, University of Jeddah, Jeddah, Saudi Arabia; ^3^Department of Biomedical Sciences, Faculty of Health and Medical Sciences, University of Copenhagen, Copenhagen, Denmark; ^4^School of Physiology, Pharmacology and Neuroscience, and Cardiovascular Research Laboratories, Faculty of Life Sciences, University of Bristol, Bristol, United Kingdom; ^5^Peng Cheng Laboratory, Shenzhen, China; ^6^Key Laboratory of Medical Electrophysiology of Ministry of Education and Medical Electrophysiological Key Laboratory of Sichuan Province, Institute of Cardiovascular Research, Southwest Medical University, Luzhou, China

**Keywords:** aging, SA node, ion channel remodeling, *I*_*CaL*_, sick sinus syndrome (brady-arrhythmia)

## Abstract

The function of the sinoatrial node (SAN), the pacemaker of the heart, declines with age, resulting in increased incidence of sinoatrial node dysfunction (SND) in older adults. The present study assesses potential ionic mechanisms underlying age associated SND. Two group studies have identified complex and various changes in some of membrane ion channels in aged rat SAN, the first group (Aging Study-1) indicates a considerable changes of gene expression with up-regulation of mRNA in ion channels of Cav1.2, Cav1.3 and KvLQT1, Kv4.2, and the Ca^2+^ handling proteins of SERCA2a, and down-regulation of Cav3.1, NCX, and HCN1 and the Ca^2+^-clock proteins of RYR2. The second group (Aging Study-2) suggests a different pattern of changes, including down regulation of Cav1.2, Cav1.3 and HCN4, and RYR2, and an increase of NCX and SERCA densities and proteins. Although both data sets shared a similar finding for some specific ion channels, such as down regulation of HCN4, NCX, and RYR2, there are contradictory changes for some other membrane ion channels, such as either up-regulation or down-regulation of Cav1.2, NCX and SERCA2a in aged rat SAN. The present study aims to test a hypothesis that age-related SND may arise from different ionic and molecular remodeling patterns. To test this hypothesis, a mathematical model of the electrical action potential of rat SAN myocytes was modified to simulate the functional impact of age-induced changes on membrane ion channels and intracellular Ca^2+^ handling as observed in Aging Study-1 and Aging Study-2. The role and relative importance of each individually remodeled ion channels and Ca^2+^-handling in the two datasets were evaluated. It was shown that the age-induced changes in ion channels and Ca^2+^-handling, based on either Aging Study-1 or Aging Study-2, produced similar bradycardic effects as manifested by a marked reduction in the heart rate (HR) that matched experimental observations. Further analysis showed that although the SND arose from an integrated action of all remodeling of ion channels and Ca^2+^-handling in both studies, it was the change to *I*_*CaL*_ that played the most important influence.

## Introduction

The sinoatrial node (SAN) is the primary pacemaker of the heart. It is situated in the superior right atrium and produces a sequence of auto-rhythmic electrical activities that control the heartbeat (Baruscotti and Robinson, 2007). Dysfunction of the sinoatrial node (SND) associated with “sick sinus syndrome” (SSS) manifests as pathological bradycardia or systolic pauses (Haqqani and Kalman, 2007), producing inadequate blood supply to satisfy the demands of the body. This condition leads to symptoms such as dizziness and syncope (Haqqani and Kalman, 2007), though the initial stages of SND may be latent and asymptomatic (Choudhury et al., 2015).

Various physiological and pathological mechanisms (intrinsic, extrinsic or a combination of the two) are responsible for SND (Semelka et al., 2013). Among them is the failure of a specific part of the SAN to achieve impulse generation (Fenske et al., 2013), or the impulse conduction from the SAN to the surrounding atrial muscle, due to genetic mutations (Butters et al., 2010; Choudhury et al., 2018) or ischemic conditions (Bai et al., 2017). Increased fibrosis and degenerative changes in physiological properties of the SAN may also cause SND (Baruscotti and Robinson, 2007; Zhang et al., 2007; Choudhury et al., 2015).

Sinoatrial node dysfunction is also associated with aging (Tohno et al., 2014; Alghamdi et al., 2018). Whilst it can occur at any age, the occurrence of SND significantly increases with age (Härtel and Talvensaari, 1975). In a retrospective research study of 277 participants with compromised bradycardia, 51% of the cases were attributed to extrinsic causes (such as adverse drug reactions, imbalance of electrolytes or acute myocardial infarction, etc.), and 49% were attributable to either intrinsic or idiopathic (Sodeck et al., 2007) conditions, such as cardiac ischemia, gene mutations, excessive training and particularly aging (Semelka et al., 2013; Choudhury et al., 2015). More than 600,000 pacemaker implants are performed worldwide each year (Lamas et al., 2000; Wood and Ellenbogen, 2002; Bai et al., 2017), of which older adults account for the largest percentage.

Experimental data from animal models (Alings and Bouman, 1993; Satoh et al., 2005; Jones et al., 2007; Moghtadaei et al., 2016; Du et al., 2017) and human studies (Opthof, 1994; Dobrev, 2009) have demonstrated an association between SND and aging. It has been shown that during the aging process, changes occur to SAN function, manifested by increased pacemaking cycle length (CL) (*i.e.*, slower heart rate) and reduced conduction of action potentials [*i.e.*, an increased sinoatrial node-atrium conduction time (SACT)]. Both of these contribute to the reduced aerobic capacity of older adults, leading to an increased incidence of abnormal pacemaking and atrial arrhythmia (Sharpe et al., 2017). Further investigations have also revealed that such aging-associated SND is related to changes in the cellular electrical properties of the SAN (see Supplementary Table 1 in the Appendix) (Congxin Huang et al., 2006; Zhang et al., 2007; Hao et al., 2011; Larson et al., 2013; Tohno et al., 2014; Huang et al., 2015; Moghtadaei et al., 2016; Du et al., 2017) in a similar way to aging-induced cellular changes in atrial and ventricular cells (Congxin Huang et al., 2006) and intercellular electrical coupling (Jones et al., 2004). A list of aging-related changes in mRNA/proteins of ion channels, Ca^2+^ handling, intercellular coupling and tissue fibrosis population of the SAN for different species is given in Supplementary Table 1 in the Appendix.

Complex and variable patterns of changes to ion channels and calcium-handling proteins have been observed in aging-induced SND rat models (Tellez et al., 2011; Hatch, 2012; Huang et al., 2016). In their study, Tellez et al. (2011) (Aging Study-1) found that aging-SND was associated with a considerable changes of gene expression in the SAN, including: an increase in the relative abundance of mRNA for many ion channels [e.g., Nav1.5, Navβ1 and Cav1.2, Cav1.3 and KvLQT1, Kv4.2 (which are responsible for *I*_*Ks*_ and *I*_*to*_)] in addition to an increase of the Ca^2+^ handling proteins, SERCA2a, that responsible for Ca^2+^-uptake process. The study also showed, a decrease in some other ion channels (e.g., in Cav3.1, NCX, and HCN1) and in the Ca^2+^ clock (RYR2) gene expressions of the SAN during aging. This implied changes of them during aging, though there is no explicit link between mRNA levels and channel activity (Brioschi et al., 2009). However, other studies (Aging Study-2) showed conflicting data, suggesting a trend of down-regulation of some ion channels (e.g., decreases of Cav1.2, HCN4, and RyR2 densities and proteins, and an increase of NCX and SERCA densities and proteins) in aged SAN cells (Hatch, 2012; Huang et al., 2016).

The ionic mechanisms that underlie the aging associated SND are as yet unclear. It is possible that SND is associated with different “patterns” of ionic and molecular remodeling mechanisms, including up-regulation or down-regulation of particular ion channels as identified in previous studies (Tellez et al., 2011; Hatch, 2012; Huang et al., 2016). The aims of the present study were to:

1. determine whether the age-induced changes in ion channels as identified in Aging Study-1 and Aging Study-2 were sufficient to account for the observed SND;
2. investigate the relative roles of particular remodeled ion channels on modulation of the characteristics of pacemaking APs, including the cycle length (CL), action potential duration (APD_50_), peak amplitude (PA), maximal upstroke velocity (dV/dt_*max*_), and maximal diastolic potential (MDP); and
3. address the question of how differently remodeled *I*_*Ca,L*_ could lead to SND.

## Materials and Methods

### Mathematical Models of Single SAN Cells

The consequence of aging-induced electrophysiological changes in ion channel currents in rat SAN cells was investigated using the model developed by Tao et al. (2011) for rat pacemaking APs. The model was based on modifications of rabbit SAN models (Zhang et al., 2000; Kurata et al., 2002) by incorporating experimental data for major ion channels obtained from rat SAN cells by Satoh (2003a, b) and Shinagawa et al. (2000). The data included aging-induced remodeling in key currents – *I*_*Ca*_,_*L*_, *I*_*Ks*_, *I*_*Kr*_, and *I*_*f*_ – which were modeled by incorporation into classical Hodgkin-Huxley formulations.

In the Tao et al. model, the membrane potential *V*_*m*_ is dependent on a set of voltage-gated ion-channel currents, exchanger currents, and ionic pump currents, in the form of the following equations (1) and (2):

(1) $$\frac{d\,V_{m}}{d\,t}=-\frac{1}{C_{m}}I{}_{t\,o\,t}$$

(2) I=tot(I+Ca,TI+Ca,LI+KrI+KsI+stI+sus  I+toI+K,AChI+fI+b,NaI+NaCaI)NaK

where *C*_*m   *_is the cell capacitance (set at 32pF); *I*_*f*_ the hyperpolarization-activated current; *I*_*Ca,L*_ and *I*_*Ca,T*_ the inward L-type and T-type Ca^2+^ currents; *I*_*Kr*_ and *I*_*Ks*_ the rapid and slow delayed rectifier K^+^ currents; *I*_*to*_ the Ca^2+^-independent transient outward K^+^ current; *I*_*st*_ the sustained inward current (carried by Na^+^); *I*_*sus*_ the sustained component of 4-AP sensitive current; *I*_*K,ACh*_ the muscarinic K^+^ channel current; *I*_*NaK*_ the Na^+^-K^+^ pump current; *I*_*NaCa*_ the Na^+^-Ca^2+^ exchanger current; and *I*_*b,Na*_ the background inward Na^+^ current. Details of the equations and parameters of the model are documented in Tao et al. (2011).

We obtained the source code of this mathematical model *via* a request to the author. The original model was developed with neural-myocyte coupling and the modulation of pacemaking by nitric oxide and cyclic GMP in response to brief sympathetic stimulations observed in hypertension. As the present study was focused on studying the effects of aging on the morphology and characteristics of the pacemaking APs, therefore the neural-myocyte coupling was removed from the model, resulting in the simulated cycle length of the pacemaking APs at 230 ms, which is slightly different to that of the original model at 257 ms, but is still within the experimental range value of the rat SAN in Shinagawa et al. (2000). Numerically, the Hodgkin-Huxley equations for the rat SAN cell model were solved with a time step of 0.01 ms, which was sufficiently small to ensure a stable numerical solution.

### Aging SAN Model

To simulate the functional impact of aging-induced changes of membrane ion channels and intracellular Ca^2+^-handling on cardiac pacemaking activity, experimental data from two independent groups of studies on aging rat SAN cells, as identified by Tellez et al. (2011) (denoted Aging Study-1); and Hatch (2012) and Huang et al. (2016) (collectively denoted Aging Study-2) for adult and older adult rat SAN were incorporated into the model Tao et al. (2011). As some of the experimental data were derived from mRNA gene expression or protein levels, their incorporation was carried out with an assumption on that there is a correlation between the gene expression or protein levels and certain ion channel current densities as we did in our previous studies (Hao et al., 2011; Choudhury et al., 2018). Both datasets were obtained from SAN cells isolated from the right atrium of Wistar Hannover rats with ages of ≥25 or ≥24 months (equivalent to about a 70-year-old human) (Tellez et al., 2011; Hatch, 2012). In both studies, cellular APs and ECGs were recorded. These data showed reductions in heart rates in the older adult rat SAN preparations by 18 (Tellez et al., 2011) and 11% (Hatch, 2012), respectively, as compared with adult preparations. Such a change in heart rate is associated with changes in cellular ion channels, as summarized in Supplementary Table 2 (see Appendix).

Changes of different ion channels in the aging condition as shown in Supplementary Table 2 were incorporated into the Tao et al. model through modifying the maximal channel conductance value of a particular ion channel equation. To simulate the effects of the aging-induced changes of the expression for RyR and SERCA, we modified the Tao et al. model which includes the Ca^2+^ handling equations from the Zhang et al. (2000) and Kurata et al. (2002) models of the rabbit SA node cells, by changing the flux of the Ca^2+^ release from the SR (*J*_*rel*_) and Ca^2+^ uptake by the SERCA (*P*_*up*_) based on scaling factors as listed in Supplementary Table 2. The equations for the SR Ca^2+^ release and SERCA Ca^2+^ uptake are as follows:

(3) Ju⁢p=Pu⁢p×[Ca  2+]i[Ca  2+]i+Ku⁢p

(4) Jr⁢e⁢l=Pr⁢e⁢l×([Ca   2+]r⁢e⁢l-[Ca   2+]s⁢u⁢b)×[Ca  2+]s⁢u⁢b2[Ca  2+]s⁢u⁢b2+Kr⁢e⁢l   2

In simulations, AP characteristics including CL (and hence HR), maximal diastolic potential (MDP), peak amplitude (PA) and action potential duration (APD_50_) were computed in adult and older adult conditions, which were compared with the relevant experimental data (Hatch, 2012; Huang et al., 2016) for validation. In order to elucidate the primary factor(s) responsible for the slower heart rate in the older adult cells, the relative impact of different remodeled ionic channels on CL were analyzed *via* two distinct methods: the inclusive, and the exclusive. With the inclusive method, all changes in the remodeled ion channels and Ca^2+^-handling due to aging were considered for the older adult condition. With the exclusive method, only a specific remodeled ion channel or subsets of all the remodeled ion channels were considered for the older adult condition, while the rest of the aging-induced changes were not included in the models. Details of ion channels considered in each case are described in the following sections.

## Results

### Effects of Ion-Channel Remodeling on Pacemaking APs

**FIGURE 1:**
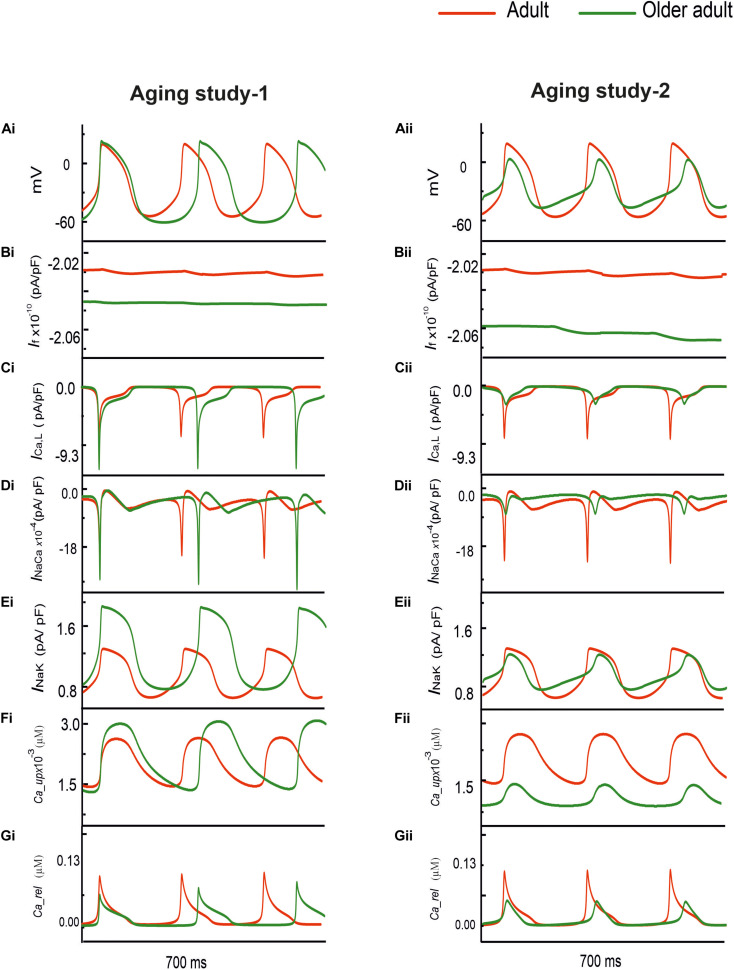
**(Ai,Aii)** Action potentials computed from rat SAN myocyte model in adult (red) and older adult (green) conditions using the Aging Study-1 **(left panels**) and Aging Study-2 **(right panels)** datasets. Simulations were conducted by using the rat SAN cell model developed by Tao et al. (2011). **(Bi–Gi,Bii–Gii)** Underlying ionic channel currents and Ca^2+^-handling during APs: *I*_*f*_, *I*_*CaL*_, *I*_*NaCa*_, *I*_*NaK*_, Ca^2+^ uptake and Ca^2+^ release.

Figure 1 shows the simulated effects of aging on the pacemaking AP of rat SAN cells based on the experimental data set of Aging Study-1 (left panels) and Aging Study-2 (right panels). Both aging datasets produced a similar consequence of aging-related bradycardia, *i.e.*, a slower pacemaking rate in the older adult compared with the adult condition, although there are dramatic differences in the underlying remodeling of membrane ion channels and the Ca^2+^-handling (see Supplementary Table 2 in the Appendix). With the use of Aging Study-1 data (Tellez et al., 2011), the CL was increased from 230 ms in the control condition to 310 ms in the older adult condition, implying a 19% decrease in pacemaking rate (HR). Similarly, using the Aging Study-2 data, an increase of the CL from 230 to 265 ms was observed, corresponding to ∼12.9% decrease in HR. Such simulated effects of aging on slowing the cardiac pacemaking APs are in agreement with those observed experimentally (Hatch, 2012; Huang et al., 2016).

Although simulations using both experimental data (Tellez et al., 2011) sets showed similar effects on the reduction of the pacemaking rate, each of which produced different changes in the characteristics of the simulated APs. In the case of Aging Study-1, the simulated pacemaking APs in the older adult condition (Figure 1Ai) presented a more hyperpolarized maximum diastolic potential (MDP) (by −12.8 mV), a greater dV/dt_*max*_ (by 60%), PA (by 20%), and APD_50_ (by 14%). These changes in AP characteristics were attributable to changes in the underlying ion-channel currents and Ca^2+^-handling, which were manifested as a decreased *I*_*f*_ (Figure 1Bi), increased *I*_*Ca,L*_ (Figure 1Ci), decreased *I*_*NaCa*_ (Figure 1Di), as well as an increased *I*_*NaK*_ (Figure 1Ei) and Ca^2+^-uptake (Figure 1Fi), but a decreased Ca^2+^ release (Figure 1Gi).

However, in the case of Aging Study-2, the simulated pacemaking APs (Figure 1Aii) presented an elevated maximum diastolic potential value (by + 9.3 mV), decreased dV/dt_*max*_ (by 64%) and PA (by 70%), as well as an abbreviated APD_50_ (by 17%). These changes were attributable to decreased *I*_*f*_ (Figure 1Bii) and *I*_*CaL*_ (Figure 1Cii), increased *I*_*NaCa*_ (Figure 1Dii) and Ca^2+^ uptake (Figure 1Fii), but decreased Ca^2+^ release (Figure 1Gii). The decrease in PA was attributable to the reduction of *I*_*CaL*_.

**FIGURE 2:**
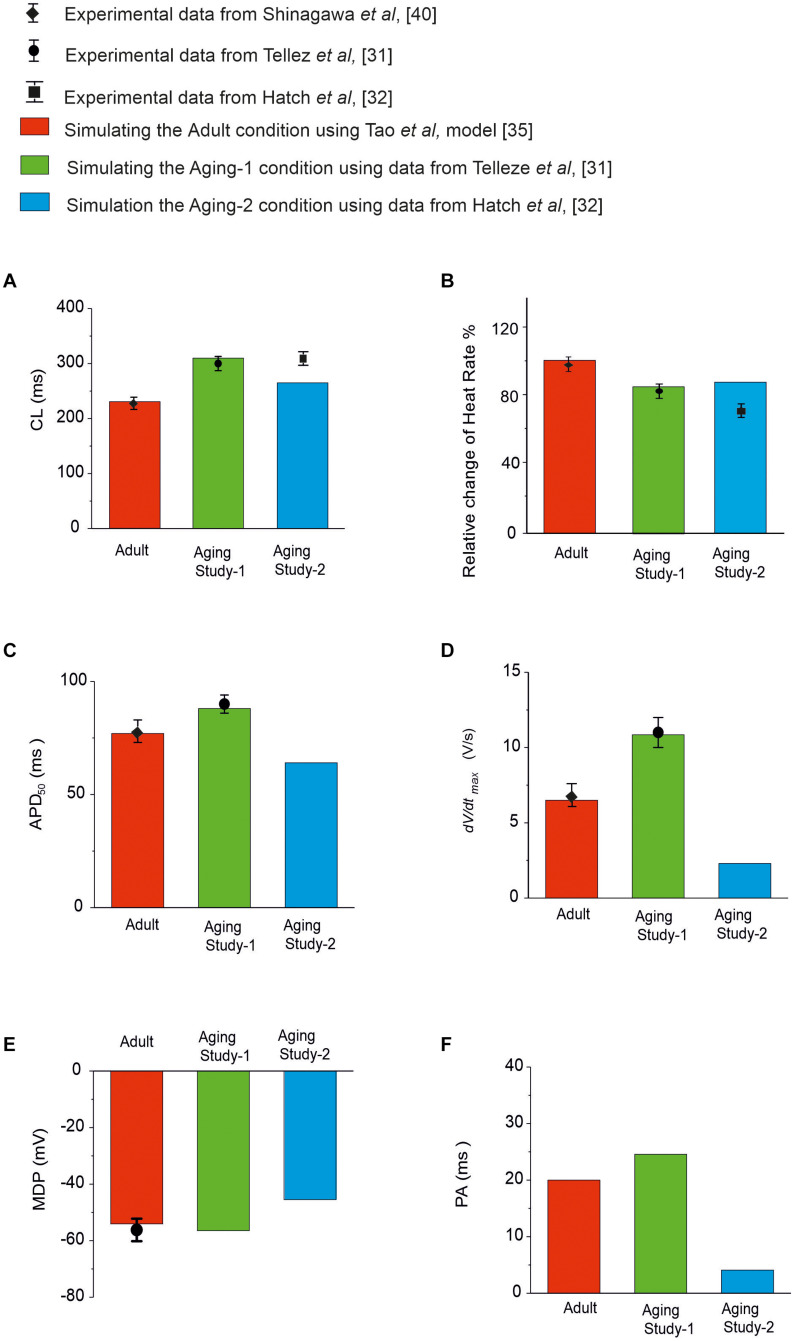
Quantitative comparison between experimental and simulation data of the functional effects of aging on the pacemaking APs and their characteristics. **(A)** CL; **(B)** HR; **(C)** APD_50_; **(D)** dV/dt_*max*_; **(E)** MDP; and **(F)** PA. Data for adult is shown in red, and from Aging Study-1 and -2 in green and blue, respectively. Experimental data with error bars, where available, are from Tellez et al. (2011); Hatch (2012), Huang et al. (2016), and Shinagawa et al. (2000), which were all from rat SAN cells.

Simulations of the aging effect on the pacemaking APs and their characteristics were validated by quantitatively comparing the simulation data to experimental data when possible. Results are shown in Figure 2 and summarized in Supplementary Table 3 in the Appendix. In the case of Aging Study-1, simulations showed about 80 ms increase in CL, leading to a 19% reduction in HR. This is quantitatively consistent with the study of Tellez et al. (2011), who observed about 30% increase in CL that corresponded to an 18% reduction in HR. We also observed increases in APD_50_ and maximal dV/dt_*max*_ in simulations, that were quantitatively consistent with the results of Tellez et al. (2011).

In Aging Study-2, our simulations showed that aging caused an increased CL by 35 ms, which corresponded to a reduction in HR of about 12.9%. This was also comparable with data from Hatch (2012), who observed a CL increase of 40% in CL; corresponding to a 30% decrease in HR. It was also comparable with the data from Huang et al. (2016), which showed an increase of the CL from 193 to 235 ms (the intrinsic HR changed from 310 to 255 bpm) with aging, corresponding to 17.7% reduction of the intrinsic HR. We also observed decreases in APD_50_, dV/dt_*max*_ and the amplitude of action potentials. However, there are no experimental data available for quantitative comparison with these values.

### Relative Role of Individual (or a Subset of) Remodeled Ion Channel(s) in Aging Bradycardia

Simulation results using the data sets from Aging Study- 1 and 2 produced similar bradycardic effects that both qualitatively and quantitatively matched to their experimental observations (although limited experimental observations for Aging Study-2 were available), despite of marked differences in their underlying remodeled ion channels and Ca^2+^-handling were observed. In order to elucidate the primary factor(s) that contributed to aging bradycardia, effects and their relative roles of individual, or a subset of, remodeled ion channels and Ca^2+^-handling on pacemaking APs were simulated and analyzed using the exclusive method. Through this method, only some specific aging-induced change(s) were considered, while other remodeling factors were omitted.

After analysis of the differences and similarities in the aging-induced remodeling patterns between Aging Study- 1 and 2, three different cases were analyzed, as shown below.

#### Case 1: Effects of Remodeled *I*_*f*_ and RyR2

**FIGURE 3:**
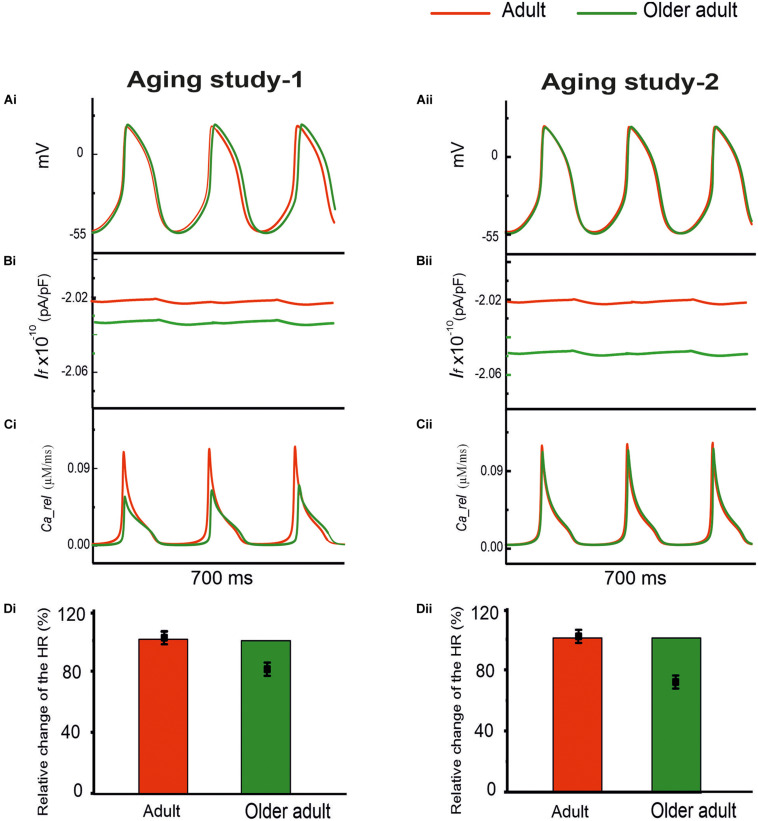
Simulated role of reduced *I*_*f*_ and Ca^2+^ release from the SR in aging bradycardia. Changes in other ion-channel currents as reported by Tellez et al. (2011) in Aging Study-1 (right panels) and by Hatch (2012) and Huang et al. (2016) in Aging Study-2 (left panels) were omitted. **(Ai,Aii)** APs. **(Bi,Bii)** Ionic current profiles for *I*_*f*_, and **(Ci,Cii)** for Ca^2+^ release, for adult (red) and older adult (green) conditions. **(Di,Dii)**: Bar charts showing the simulated age-dependent relative change in HR for this case; data-points with error bars show the experimental HR reduction due to aging.

Both group aging studies showed a down-regulation of HCN channels in mRNA for aging study-1 and in protein expression for aging study-2. For RyR Ca^2+^ release channels, also, presented a reduction of in mRNA expression in aging study-1, in protein expression and expression density of aging-2. Therefore, in Case 1, simulations were conducted to evaluate the effects of a reduced *I*_*f*_ by 16% in Aging Study-1 [this value was based on the mRNA expression data in Tellez et al. (2011) study], and 30% in Aging Study-2 [this value was based on the data of Huang et al. (2016) study], and RyR Ca^2+^ release from the sarcoplasmic reticulum (SR), by 80% in Aging Study-1 [this value was obtained from the mRNA expression data in Tellez et al. (2011) study], and 24% in Aging Study-2 [this value was based on the protein expression density data in Hatch (2012) study], on the pacemaking APs. Results in Figure 3 show the computed APs for the control (adult) and for the older adult conditions based on Aging Study-1 [left panels (Ai)] and Aging Study-2 [right panels (Aii)]. During the time course of APs, the time traces of *I*_*f*_ and Ca^2+^ release flux from the SR are also shown in panels (Bi) and (Bii), and (Ci) and (Cii), for the adult and older adult conditions, respectively.

With both data sets, reduced *I*_*f*_ and Ca^2+^ release from the SR had a limited effect on slowing down pacemaking rate. With a combined action of reduced *I*_*f*_ and Ca^2+^ release, the HR was reduced with the dataset from Aging Study-1, but unchanged with the dataset from Aging Study-2.

Simulation results from both datasets suggested a limited role of combined action of remodeled *I*_*f*_ and Ca^2+^ release in the bradycardia. Further analysis was conducted to analyze the individual role of a wide-range reduction of *I*_*f*_ or SR Ca^2+^ release. When *I*_*f*_ reduction alone was considered from 16% (Aging Study-1) to 30% (Aging Study-2), there was no noticeable change in spontaneous APs (data not shown). This result was in agreement with the results of Bers (2001), who observed that *I*_*f*_ played only a minor role in primary and central SAN cells, though a critical role in peripheral SAN cells.

When a reduced SR Ca^2+^ release alone was considered, either by 80% (Aging Study-1) or by 24% (Aging Study-2), there was a small increase in the measured CL. With an 80% reduction in the RyR2 alone, as suggested by Aging Study-1, CL was increased by 2.45%, while with a 24% reduction in the RyR2 alone as seen in Aging Study-2, no noticeable change in the CL was observed (data not shown).

To test whether or not the simulated consequences of RyR2 reduction by 80% (Aging Study-1), and 24% (Aging Study-2) are model-dependent, the Maltsev and Lakatta (2009) model for the rabbit SAN cells was also used. Results are shown in Supplementary Figure 1. It was found that a 24% reduction of the RyR2 had little effect on reducing the pacemaking rate, while 80% reduction of RyR2 produced a small, but notable change of the APs, manifested by an increased CL (by 5.2%), elevated MDP and less steep DD phase, leading to an increased time taken to producing the successive peacemaking APs. The simulation results using the Maltsev and Lakatta (2009) model for the rabbit SAN cells matched the findings using the Tao et al. model for the rat SAN cells in showing that the aging-induced change in RyR made a limited contribution to aging-associated modulation of SAN spontaneous APs.

The time traces of Ca^2+^ release flux during the APs are shown in panels (Ci) and (Cii) for the adult (red) and older adult (green) conditions (with a combined action of reduced *I*_*f*_ and Ca^2+^ release). Panels (Di) and (Dii) compare the simulation and experimental results of relative changes in HR when the age-induced remodeling of *I*_*f*_ and RyR2 alone were considered.

#### Case 2: Effects of Remodeled *I*_*Ca,L*_, *I*_*NaCa*_, and SERCA2a

Both the experimental datasets showed changes to *I*_*Ca,L*_, *I*_*NaCa*_ and Ca^2+^ uptake from the SR with aging, but with conflicting findings. In Aging Study-1, up-regulation of *I*_*Ca*_,_*L*_ and Ca^2+^ uptake, but down-regulation of *I*_*NaCa*_, were observed; whilst in Aging Study-2, down-regulation in *I*_*Ca*_,_*L*_ and Ca^2+^ uptake, but an up-regulation of *I*_*NaCa*_, were shown.

In order to investigate the role of this conflicting subset of aging-induced remodeling shown by the two sets of data, further simulations were conducted by incorporating the altered *I*_*Ca,L*_, *I*_*NaCa*_ and Ca^2+^ uptake into the model to investigate their contribution to the aging bradycardia.

**FIGURE 4:**
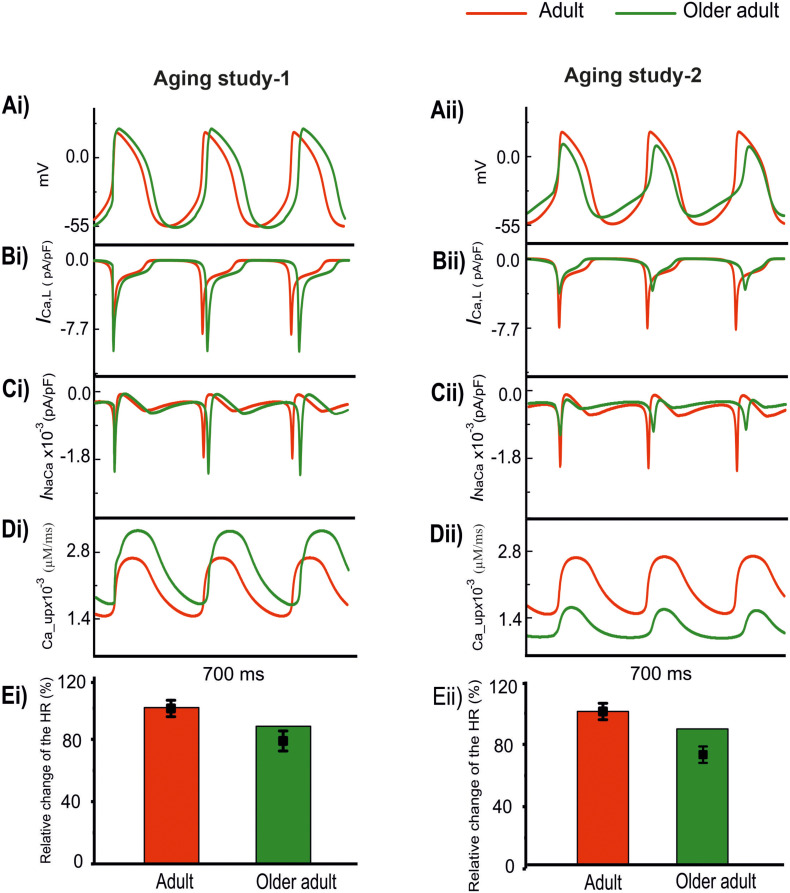
Effect of remodeled *I*_*Ca,L*_, *I*_*NaCa*_, and Ca^2+^ uptake on pacemaking APs (red: adult; green: older adult). Other ion channel changes, as seen by Tellez et al. (2011) in Aging Study-1 **(right panels)**, and by Hatch (2012) in Aging Study-2 **(left panels)**, were omitted in simulations. **(Ai,Aii)** APs. **(Bi,Bii)** *I*_*Ca,L*_. **(Ci,Cii)** *I*_*NaCa*_. **(Di,Dii)** Ca^2+^ uptake. **(Ei,Eii)** Relative HR and its reduction. Data-points with error bars show the experimental data.

Results are shown in Figure 4 for the computed APs (Ai and Aii) in the adult and older adult conditions, as well as the underlying time courses of *I*_*Ca,L*_ (Bi, Bii), *I*_*NaCa*_ (Ci, Cii), and Ca^2+^-uptake (Di, Dii) based on data of Aging Study-1 (left panels) and -2 (right panels). Figure 4Ai shows the simulated pacemaking APs based on data from Aging Study-1, with considerations only of an increase of *I*_*Ca*_,_*L*_ by 25%, Ca^2+^ uptake by 15%, and a decrease in *I*_*NaCa*_ by 6% for the older adult condition [data based on the mRNA expression data in Tellez et al. (2011) study]. As compared with the adult condition, the computed CL increased by 23.4% (*i.e.*, a decrease in HR of 11%), and this was accompanied by changes in other AP characteristics, including an increase in PA of 17.5% and in APD_50_ of 23.4%. In this case, the observed changes in CL and AP characteristics were comparable with those obtained when all remodeled factors were considered, as well as close to experimental observations (Tellez et al., 2011). The results seem to suggest that the aging-induced up-regulations of *I*_*Ca*_,_*L*_ and Ca^2+^-uptake, and down-regulation of *I*_*NaCa*_, are sufficient to account for the aging bradycardia seen experimentally in Aging Study-1.

Figure 4Aii shows simulated APs based on data from Aging Study-2 with a consideration of a decrease of *I*_*Ca*_,_*L*_ by 50%, Ca^2+^-uptake by 29%, and an increase of *I*_*NaCa*_ by 42% [data based on the protein expression density data in Hatch study (Hatch, 2012) as shown in Supplementary Table 2]. As compared with the APs in the adult condition, the implemented changes also reproduced bradycardia, resulting in a 9% increase in CL (i.e., a 7% HR decrease), which was accompanied by other changes in AP characteristics. These included an elevated MDP by 6 mV, a reduction of PA by 55% and an APD_50_ shortening by 14%. Those observed changes in APs were also close to the experimental observations of Hatch (2012), which implied that the subset of remodeled *I*_*Ca*_,_*L*_, *I*_*NaCa*_, and Ca^2+^ uptake, as seen in Aging Study-2, were also sufficient to produce the aging-related bradycardia.

The use of this subset of data from either Aging Study-1 or Aging Study-2 produced bradycardia, though there are clear differences between the two. In Aging Study-1, bradycardia was associated with an increased *I*_*Ca*_,_*L*_ (Figure 4Bi) and SR Ca^2+^ uptake (Figure 4Ci), and a decreased *I*_*NaCa*_ (Figure 4Di). In contrast, in Aging Study-2 it was associated with a decreased *I*_*Ca*_,_*L*_ (Figure 4Bii) and decreased SR Ca^2+^ uptake (Figure 4Cii), and an increased *I*_*NaCa*_ (Figure 4Dii). Quantitatively, the computed HR reductions in the older adult condition were close to the experimental data of Tellez et al. (2011) and Hatch (2012) for Aging Study-1 (Figure 4Ei) and Aging Study-2 (Figure 4Eii), respectively.

**FIGURE 5:**
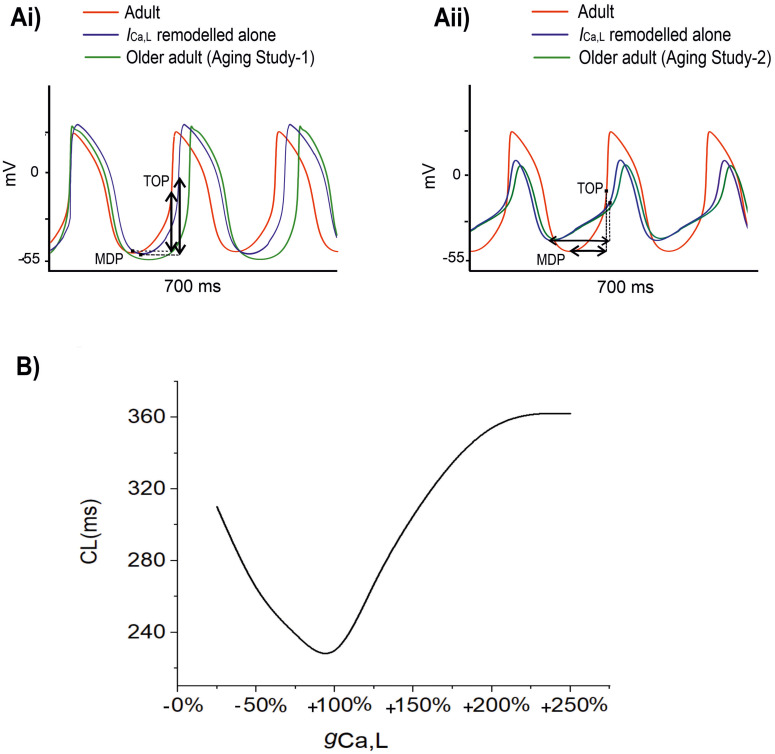
**(A)** Simulated APs when the age-related remodeling of *I*_*Ca,L*_ alone (blue) was considered [other ion channel and Ca^2+^ handling changes, as seen by Tellez et al. (2011) in Aging Study-1 (right panels), and by Hatch (2012) in Aging Study-2 (left panels), were ignored]. The obtained APs were compared with those of adult (red) and those with all ion channel and Ca^2+^ handling remodeling were considered (green). **(B)** Relationship between CL and increase (positive direction) or decrease (negative direction) in g_*CaL*_. It demonstrates that CL increases with either an increased or a decreased g_*CaL*_ compared to the reference value of 100%. MDP: the maximal diastolic membrane potential. TOP, the take-off potential at which a line drawn from the MDP along the diastolic depolarization time course intersects with a vertical line drawn from the AP overshoot.

Figure 5 shows the results when aging-related remodeling of *I*_*CaL*_ in a range based on the study of Tellez et al. (2011) and Hatch (2012) was considered. In Aging Study-1 (Figure 5Ai), the 25% up-regulated *I*_*Ca,L*_ increased the peak amplitude of the AP from 20 to 25 mV and prolonged the APD_50_ from 77.06 to 95.11 ms, which slowed down the time course for repolarization and consequently led to a slowing down in the pacemaking as seen in aging bradycardia when all remodeled ion channels were considered in Aging Study-1. It also produced a more hyperpolarized maximal diastolic membrane potential, which led to an increased voltage difference between the maximal diastolic membrane potential, MDP, and the take-off potential (TOP), at which a line from the MDP along the diastolic depolarization time course intersects with a vertical line drawn from the AP overshoot. This contributed to increased time taken to generate a successive action potential, though the depolarization *I*_*NaCa*_ current during the diastolic depolarization (DD) phase was increased. Overall, this led to an increased time interval between two successive APs (i.e., CL), leading to a slowed heart rate (see Table 1). Figure 5B shows how both increase and decrease of the *I*_*Ca,L*_ through changing the gCaL can affect the measured CL leading to increase the pacemaking.

**TABLE 1:** Quantitative analysis of the role of age-related remodeling of *I*_*Ca,L*_ alone in bradycardia as compared to the case when all remodeling were considered.

APS characteristics	Adult	Aging study 1		Aging study 2	
		+*25% I*_*Ca,L*_ remodeled alone	All ion-channel remodeling	−50% *I*_*Ca,L*_ remodeled alone	All ion-channel remodeling
dV/dt_*max*_ (V/s)	6.50	7.22	10.85	3.20	2.31
MDP (mV)	−54.01	−55.02	−56.84	−47.00	−45.70
PA (mV)	20.00	25.00	24.58	6.61	9.5
APD_50_ (ms)	77.06	95.11	88.00	57.77	64.00
CL (ms)	230.00	267.50	310.44	260.00	265.22

In Aging Study-2 (Figure 5Aii), a decreased *I*_*Ca,L*_ also produced bradycardia. In this case, the decreased *I*_*Ca,L*_ was associated with a decreased AP peak amplitude from 20 to 6.61 mV and abbreviated APD_50_ from 77.06 to 57.77 ms. Both accelerated the repolarization process but produced an incomplete repolarization as a consequence of reduced AP amplitude and abbreviated APD_50_. Consequently, an elevated MDP was produced, leading to a reduced depolarization current in the DD phase (such as *I*_*NaCa*_), which slowed down the time course between the MDP and the take-off potential, leading to an increased time interval between two successive pacemaking APs, resulting in bradycardia that was also comparable to the case when all remodeled ion channels were considered in Aging Study-2. Figure 5B shows how either an increase or a decreased *I*_*Ca,L*_ through changing g_*CaL*_ modulates the measured CL leading to bradycardiac pacemaking.

In Aging Study-1, when the age-related increase in SERCA2a (by 15%), or the decrease in NCX (by 6%) alone was considered, negligible effects in modulating the pacemaking APs was observed. Similarly, in Aging Study-2, when a decrease in SERCA2a (by 29%) or an increase in NCX (by 42%) alone was considered, their effect on the pacemaking APs was also limited.

These simulations implied that based on experimental data of Aging Study- 1 and 2 the altered *I*_*Ca,L*_ produced notable effects on the modulation of the pacemaking AP profiles and rates, leading to bradycardia, though through different actions of either up-regulation or down-regulation of it.

#### Case 3: Effect of Remodeled *I*_*Kr*_, *I*_*Ks*_, *I*_*to*_, *I*_*NaK*_, and *I*_*Ca,T*_ (Specific for Dataset 1)

Aging Study-1 also found changes in other ion channels, including *I*_*Kr*_, *I*_*Ks*_, *I*_*to*_, *I*_*Nak*_, and *I*_*Ca,T*_, which were absent in Aging Study-2. Further simulations were conducted to investigate the effects of such a subset of remodeled ion channels on aging bradycardia. In simulations, we considered the integral action of changes to *I*_*Kr*_ (by a 6% reduction), *I*_*Ks*_ (by a 25% increase), *I*_*to*_ (by a 60% increase), *I*_*Na*__*K*_ (by a 50% increase), and *I*_*Ca,T*_ (by a 12% decrease).

**FIGURE 6:**
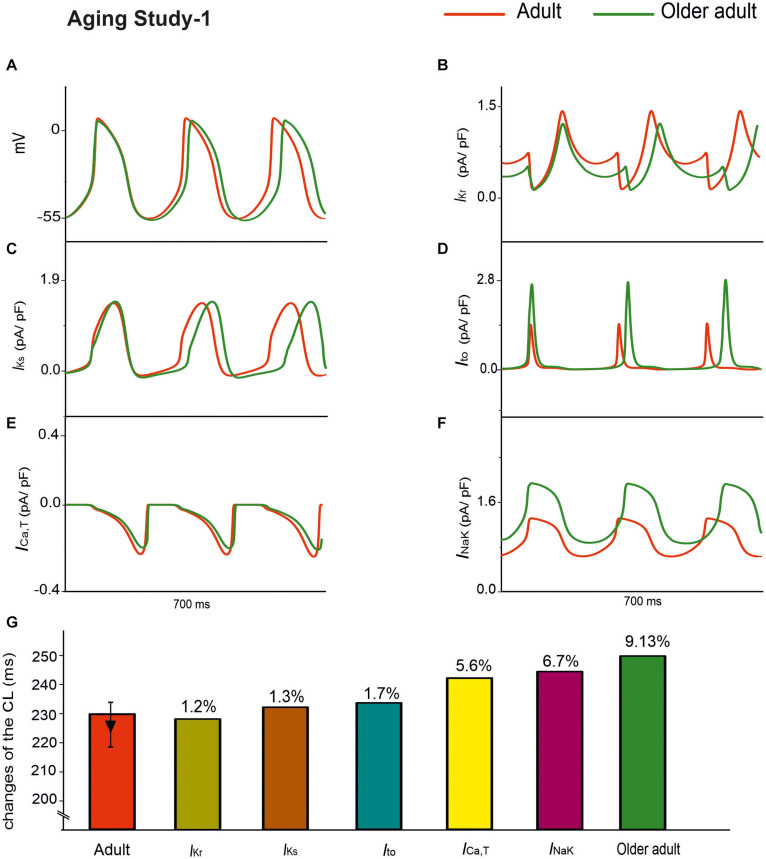
APs for the adult and older adult conditions with the subset of changes to the ion-channel currents involved in Aging Study-1, namely *I*_*Kr*_, *I*_*Ks*_, *I*_*to*_, *I*_*Ca,T*_, and *I*_*NaK*_ **(A)**. Panels **(B–F)** show the time traces for the relevant ion-channel currents, namely *I*_*Kr*_, *I*_*Ks*_, *I*_*to*_, *I*_*Ca,T*_ and *I*_*NaK*_ in the adult and older adult conditions. **(G)** Illustrates the role of the individual remodeled ionic channels on CL values.

Simulation results of APs for the adult and older adult conditions are shown in Figure 6A. In this case, the remodeled ion channels produced a 9.13% increase in CL (i.e., a 6.9% decrease in HR). It also produced changes in other AP characteristics, including a more hyperpolarized MDP (by −3 mV), and a prolonged APD_50_ (by 26 ms).

Panels B–F in Figure 6 show the time traces of the relevant ion-channel currents for *I*_*Kr*_, *I*_*Ks*_, *I*_*to*_, *I*_*Ca,T*_, and *I*_*NaK*_ in the adult and older adult conditions. It was shown that during APs, the decreased of the peak amplitude might be attributable to the change in *I*_*to*_ and *I*_*K*__*s*_; the more hyperpolarized MDP might be attributable to the increased *I*_*NaK*_; the prolonged APD_50_ was attributable to the decreased *I*_*Kr*_; and the decreased DD phase was partially attributable to the integral action of the decreased *I*_*Kr*_ and *I*_*Ca,T*_. The prolonged APD_50_ slowed down the repolarization phase and, together with the slowed DD depolarization phase and the increased time interval required for membrane potential to reach the take-off potential from the MDP, resulted in a slowed HR.

The individual roles of each remodeled *I*_*Ks*_, *I*_*to*_, *I*_*NaK*_, and *I*_*Ca,T*_ ion channels were also simulated, generating a 1.3, 1.7, 6.7, and 5.6% change in CL, respectively, as shown in Figure 6G. The simulation showed that the remodeled *I*_*NaK*_ and *I*_*Ca,T*_ currents alone made notable contributions to an increased CL, in which the remodeled *I*_*Kr*_ current was responsible for the increase in APD_50_, slowing the repolarization phase of the APs.

## Discussion

The main findings of this study are the following:

(1) heart-rate reduction in aged rat sinoatrial myocytes can be sufficiently accounted for by the age-induced ionic-current and Ca^2+^-handling remodeling of *I*_*Ca*_,_*L*_, *I*_*f*_, *I*_*NaCa*_, SERCA2a, and RyR;
(2) whilst all remodeled channels and Ca^2+^-handling contribute to the heart-rate reduction in the older adult, the remodeled *I*_*Ca*_,_*L*_ plays the most significant role; and
(3) remodeled *I*_*Ca,L*_, either up-regulated or down-regulated by aging, leads to a similar bradycardia effect.

### Mechanism Underlying Age-Related SAN Dysfunction

Possible mechanism(s) underlying the age-related SAN dysfunction were investigated by both the inclusive and exclusive methods in simulations. With the inclusive method, when all remodeling of ion channels and Ca^2+^ handling identified in Aging Study-1 (Tellez et al., 2011) and Aging Study-2 (Hatch, 2012; Huang et al., 2016) were considered, a similar bradycardic effect was produced, which was manifested by a slower pacemaking rate of APs in the older adult as compared with the adult rat SAN cell. This observation suggested that the identified age-related changes in ion channels (*I*_*Ca*_,_*L*_, *I*_*f*_, *I*_*NaCa*_) and Ca^2+^-handling (SERCA2a and RyR) in both studies were adequately responsible for the experimentally observed age-related changes in APs, which were consistent to the experimental data of the two studies. Note that, though a similar bradycardic effect was produced, changes in some other AP characteristics, such as the maximal upstroke velocity (dV/dt_*max*_), AP amplitude and duration (APD_50_), were different between the two cases of simulations. While the computed dV/dt_*max*_ and APD_50_ in the older adult condition were increased in the case of Aging Study-1, they were decreased in the case of Aging Study-2.

### Role of *I*_*f*_ and Ca^2+^ RyR Release

The relative roles of aging-remodeled *I*_*f*_ and Ca^2+^-RyR release in bradycardia was investigated by using the exclusive method. When down-regulations of *I*_*f*_ and Ca^2+^ RyR release were considered for the older adult condition as seen in both Aging Studies 1 and 2, no apparent effect was observed on modulating the APs. This suggested a limited role of remodeled *I*_*f*_ and Ca^2+^ RyR release in generating the age-related bradycardic effect.

A limited role of *I*_*f*_ in aging bradycardia in slowing down the pacemaking APs as seen in the present study is consistent with the experimental observation of Shinagawa et al. (2000), who reported that *I*_*f*_ activated at a very negative threshold, −90 mV, and is little activated under the normal AP threshold range (−70 to −50 mV). As the Tao et al. model was developed for the primary pacemaking cells of the rat SAN, which has a MDP at around −55 mV, at which the activated I_*f*_ is small, and therefore contributes only slightly to the pacemaking APs of the rat SAN cells. Considering the regional differences in cellular electrophysiological properties of SAN between central and peripheral regions, including the MDP and ion channel current densities as seen in the rabbit heart (Zhang et al., 2000), it is expected that the aging-induced I_*f*_ remodeling may have a greater effect in modulating the pacemaking APs in peripheral cells as compared to the results seen in the present study using the Tao et al. model for the central SAN cells. Unfortunately, a lack of rat SAN experimental data in this regard precluded investigation of this possibility in this study. The issue certainly warrants future investigation when experimental data on the regional differences in cellular electrophysiological properties of the rat SAN become available.

In both data sets of Aging Studies 1 and 2, substantial down-regulation of RYR2 in the SAN during the aging process was observed, which was believed to be responsible for a reduced heart rate in the aged rat (Tellez et al., 2011). However, in our simulations, a reduction of SR Ca^2+^ release in the range from 30 to 80% as observed in the two experimental studies showed only limited effects in slowing down the heart rate. A complete block of Ca^2+^ release from the SR only produced a 3.5% increase in CL. This finding was in agreement with previous experimental observations, which showed that the effect of SR Ca^2+^ release on pacemaker activity in adult rat central SAN cells was small due to the poor development of SR in these cells (Tao et al., 2011).

There has been an ongoing debate as to the leading mechanism responsible for the pacemaking activity in the SAN (Lakatta and DiFrancesco, 2009). One theory is the role of the membrane clock, in which *I*_*f*_ plays an important role (DiFrancesco, 1993; Accili et al., 2002). The other is that the Ca^2+^ clock plays an important role, by which SR pumping kinetics are thought to regulate spontaneous beating in rabbit SAN (Maltsev and Lakatta, 2009). The rate of SR replenishment defines the cycle length of each natural beat. Its occurrence is based on the cytosolic Ca^2+^ availability and SERCA2a activity due to RYR2 release flux and Cav1.2 influx, as described by Vinogradova et al. (2000). Even though the SR’s role in initiating an AP is debatable and may be species dependent (DiFrancesco, 1993; Accili et al., 2002; Lakatta and DiFrancesco, 2009; Maltsev and Lakatta, 2009; Li et al., 2013), there are distinct changes in SR proteins that are clearly related to aging.

### Role of *I*_*Ca*_,_*L*_, *I*_*NaCa*_ and SR Ca^2+^ Uptake

There are contradictory changes in *I*_*Ca,L*_, *I*_*NaCa*_ and Ca^2+^ uptake from the SR between the two group datasets of the aging studies. In order to understand their relative contribution(s) to the age-related bradycardia, the exclusive method was used to investigate the collective effect of the three factors as well as their individual role. It was shown that whilst this subset of remodeling factors collectively are attributable to the bradycardic effect, it was the remodeling of *I*_*Ca,L*_ that played a primary role. In both datasets, when a down-regulation or up-regulation of *I*_*CaL*_ alone was considered, a dramatic bradycardic effect was observed, which was comparable to the case when all ion channel and Ca^2+^ handling remodeling were considered. An increase in the pacemaking CL with a decrease of the channel conductance of *I*_*CaL*_ has been shown before by Kurata et al. in the rabbit SAN cell model (Kurata et al., 2002). In the present study, we expanded their observation by showing that not only the decrease, but also the increase of I_*CaL*_ can cause slowing down in the pacemaking APs as manifested by an increase in the CL.

**FIGURE 7:**
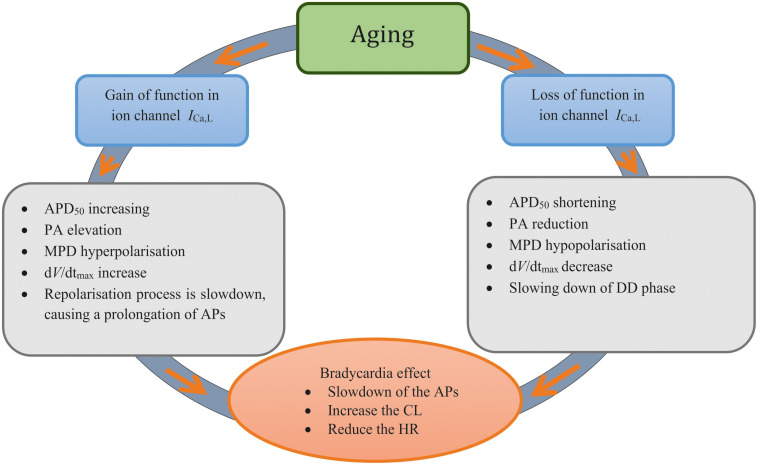
Diagrammatic representation to summarize the two ‘ways’ of remodeled *I*_*Ca,L*_ Investigated in this study and the mechanisms associated with each process.

Our results highlight the importance of the remodeled *I*_*Ca*_,_*L*_ to generate aging-related bradycardia (see Figure 7). This is consistent with a previous study, which showed that *I*_*CaL*_ was a critical pacemaking current in adult rat SAN, as demonstrated by spontaneous beating being halted by Ca^2+^ antagonists (Hatch, 2012). In simulations, we also observed a complete *I*_*Ca,L*_ block abolished the pacemaker activity (data not shown), which is consistent with the experimental observation. When remodeling in SERCA2a (by 29%) or NCX alone was considered, limited effects on pacemaking APs were observed as seen in previous studies (Katz and Miledi, 1968; Szentesi et al., 2004). Collectively, our simulation results implied that the remodeled *I*_*Ca,L*_ has the most significant influence on slowing down of the pacemaking rates in the older adult rat SAN myocytes.

### Role of Remodeled *I*_*K*_, *I*_*to*_, *I*_*Ks*_, *I*_*NaK*_, and *I*_*Ca,T*_

The role of each individual remodeled ion channel of *I*_*K*__*r*_, *I*_*to*_, *I*_*Ks*_, *I*_*NaK*,_ and *I*_*Ca,T*_, as seen in Aging Study-1, was also investigated. The functional effect of these remodeled ion channels prolonged the time course of APs, leading to a reduced pacemaking rate, some of which was associated with a secondary action. For example, the reduction in *I*_*Kr*_ prolonged the APD_50_, therefore slowing down the repolarization phase of the APs. In the adult rat SAN model, *I*_*Kr*_ is considered to be another important pacemaking current. It has been observed that during diastole, *I*_*Kr*_ is slowly deactivated and the decreased outward current enables self-activated depolarization. Therefore, in combination with other currents, remodeling of this current might play a secondary role in reducing the HR of the aged SAN (Talano et al., 1978; Hagiwara et al., 1988; Zhou and Lipsius, 1994; Lei et al., 2002; Tellez et al., 2011). Decreased spontaneous activity and sinus arrest were observed in rat SAN cells when *I*_*Kr*_ was completely blocked by E-4031 (Mitsuiye et al., 2000).

#### Are Their Multiple Causes of Aging-Dependent Sinus Node Dysfunction?

The differences between Aging Study-1 and Aging Study-2 could be the result of mRNA changes not reflecting changes in protein or erroneous protein measurement. Alternatively, the differences between the two studies could reflect different remodeling patterns to age-dependent sinus node dysfunction. The literature on age-dependent sinus node dysfunction is conflicting with different studies reporting different changes (Lamas et al., 2000; Hao et al., 2011; Moghtadaei et al., 2016). Once again the differences could be the result of shortcomings in the methodologies used, but they could be genuine. Genuine differences could be the result of the use of different species in different studies. There are other possible reasons. A clue comes from the study of Moghtadaei et al. (2016) which shows that in aged mice the sinus node dysfunction depends on the frailty of the mouse. Therefore, it is possible that in the aged animal the nature of sinus node dysfunction depends on co-morbidities. For example, in a study of age-dependent sinus node dysfunction in the rat, we noted that the old male rats were obese (Yanni et al., 2010). We have independently shown that obesity in the rat causes molecular remodeling of ion channels and sinus node dysfunction (Linscheid et al., 2019; Webb et al., 2017, 2018). Therefore, the differences between studies could be the result of co-morbities which vary in different studies. In summary, it is not proven but it is feasible that there are multiple causes of sinus node dysfunction in the old animal. This computer modeling study shows that it is at least feasible that the ion channel remodeling in Aging Study-1 and Aging Study-2 causes sinus node dysfunction. There is now a need for further experimental studies of age-dependent sinus node dysfunction taking into account species, strain, gender, age, frailty and co-morbidities – any or all of these may affect sinus node function.

## Limitations of the Study

The rat SAN model used in our simulations had certain inherent limitations, as described previously (Tao et al., 2011). Our simulation incorporated age-induced changes in ion channels and intracellular Ca^2+^ handling from two independent studies into the rat SAN model (Jones et al., 2004; Tellez et al., 2011; Hatch, 2012). The data were derived from mRNA gene expression or protein levels whenever experimental data on their kinetic or current densities in the SAN cell were unavailable. Mathematical modeling of the cardiac action potential ideally should be based on electrophysiological (usually patch clamp) recording of ionic currents obtained under physiological conditions. However, when such data are incomplete or absent, measurements of ion channel protein levels can be used as they may be used as a rough measure of ion channel density. Implicit assumptions of this approach are a direct relationship between expression levels of transcript levels or membrane proteins and the measured macroscopic channel conductance, and that relative change of expression levels may reflect changes in the macroscopic channel conductance. Whilst, such assumptions have limitations because a direct correlation between the gene expression or protein levels for certain ion channel subunits and their current densities cannot be guaranteed, this is a secondary approach in cases where direct current measurement data are missing. For example, with such an assumption, data on the expression of ion channels in the human SAN obtained by the quantitative polymerase chain reaction method, especially the ratio of the expression level between the SAN and human atrial cells have been successfully used to develop a computer model for the human SAN from the human atrial cell model (Yanni et al., 2010). Moreover, this approach has also been used effectively in additional modeling studies (Hao et al., 2011; Choudhury et al., 2018), and some computer modeling of action potentials (Chandler et al., 2009; Benoist et al., 2011; Abd Allah et al., 2012; Temple et al., 2016; D’Souza et al., 2020) have been done based on the RT-qPCR measurement of ion channel mRNA levels that often reflect protein levels (Tellez et al., 2006; D’Souza et al., 2014, 2015). In the present study, we followed the same approach as implemented by these prior studies (Yanni et al., 2010; Hao et al., 2011; Choudhury et al., 2018) and successfully reproduced the bradycardia effect in the model of Aging Study -1 which is comparable to experimental data, validating again the use of this approach.

Our results have shown that the aging-induced remodeling in I_*CaL*_ plays an important contribution to the aging bradycardia. It is known that L-type Ca^2+^ channels are multi-subunit complexes formed by different isoforms, including Cav1.2 and Cav1.3, which have different kinetics that influence their contribution to SAN pacemaking (Zuccotti et al., 2011). It would therefore be informative to further analyze their relative contributions to aging-associated bradycardia. However, due to a lack of available voltage clamp data on the activation, inactivation and channel kinetics of the two distinct I_*CaL*_ components from the rat SAN cells, it was possible in the model to consider only a general form of I_*CaL*_, without differentiating effects on the two specific isoforms. The relative roles of the two I_*CaL*_ components in the aging bradycardia warrants further investigation in future, when suitable experimental data become available.

Finally, the results of this study were determined at the single-cell level. Consequently, other factors associated with aging, such as remodeling the electrical coupling between cells via the gap junction (Huang et al., 2015), or connexin, and the possible incidence of fibrosis (Baruscotti and Robinson, 2007; Semelka et al., 2013), might also play a specific role in production of the bradycardia effect that is more pronounced at the tissue level. Nevertheless, without considering age-induced changes in the connexins and fibrosis, such changes in various ionic channel conductances can nevertheless increase CL and reduce HR in a manner that is quantitatively comparable with experimental data.

## Conclusion

In this study, the mechanism underlying the age-related SAN dysfunction in a commonly used model species was elucidated. Our results suggest that while it is an integral action of all remodeled ion channels and Ca^2+^ handling, the remodeled *I*_*Ca*_,_*L*_, either via a gain or loss of function, contributes primarily to age-related bradycardia. Although further work is required to establish the extent to which these findings also apply to the human SAN, aging-related bradycardia appears able to be linked to different remodeling of *I*_*Ca,L*_, which may ultimately have utility for clinical treatment strategies.

## Data Availability Statement

The original contributions presented in the study are included in the article/Supplementary Material, further inquiries can be directed to the corresponding author/s.

## Author Contributions

HZ conceived the study and contributed to data analysis and interpretation, supervision, and manuscript writing. AA conducted experiments, collected the data, and contributed to data analysis and interpretation and manuscript writing. JH contributed to data analysis and interpretation and manuscript writing. MB contributed to data analysis and interpretation and manuscript writing. All authors contributed to the article and approved the submitted version.

## Conflict of Interest

The authors declare that the research was conducted in the absence of any commercial or financial relationships that could be construed as a potential conflict of interest.
