# Supplementary material for: Cardiac Pacemaker Dysfunction Arising From Different Studies of Ion Channel Remodeling in the Aging Rat Heart
Source: Front Physiol. 2020 Dec 3;11:546508. doi: 10.3389/fphys.2020.546508 (PMC7744970; doi:10.3389/fphys.2020.546508)
Supplement: Supplementary file 1 [file Table_1.docx]

# Appendix

# Cardiac Pacemaker Dysfunction Arising from Different Studies of Ion Channel Remodelling in the Ageing Rat Heart

Aaazh. M. Algamdi^a,c^ , Mark R. Boyett^e^, Jules C. Hancox^a,d^, Henggui Zhang ^a,b,f*^

^a^ Biological Physics Group, Department of Physics and Astronomy, The University of Manchester, Manchester, UK

^b^ Peng Cheng Laboratory, Shenzhen, China

^c^ School of Physics, Faculty of Science, Jeddah University, Jeddah, Saudi Arabia

^d^ School of Physiology, Pharmacology and Neuroscience, and Cardiovascular Research Laboratories, School of Medical Sciences, University of Bristol, Bristol, UK

^e^ Department of Biomedical Sciences, Faculty of Health and Medical Sciences, University of Copenhagen, København, 2200, Denmark

^f^ Key Laboratory of Medical Electrophysiology of Ministry of Education and Medical Electrophysiological Key Laboratory of Sichuan Province, Institute of Cardiovascular Research, Southwest Medical University, Luzhou 646000, China

***Corresponding author at: henggui.zhang@manchester.ac.uk**

**Table 1**. Experimental studies of ageing-induced changes in mRNA/proteins of ion channel and Ca^2+^ handling in the SAN of different species. Though there are some species differences, Ageing Study-1 [31] and Ageing Study-2 [32,33] data represent some common changes in mRNA/proteins of ion channels and Ca^2+^ handling including up-regulation of Cav1.2 from guinea-pig [19] and down-regulation of HCN subunits [25,28,33], Cav1.2 and Cav3.1 [28,32]. Up-regulation of Kv4.2 was also observed in mice [28] and rat [31].

| **Method** | **Isoforms** | **Species** | **Reference** |
| --- | --- | --- | --- |
| Fibrosis accompanied by ion channel remodelling measured using relative abundance of mRNA measurement | ↑ Collagen levels, ↑ fibroblast levels, ↑ TGF-β_1_  expression of Na^+^ channel subunits (↓ Nav1.5/ (↑ Nav1.1)  expression of HCN channel subunits (↓ HCN1/ ↓ HCN2)  K^+^ channel transcripts, including ↓Kv1.2, ↓Kv1.4, ↓Kv1.5, ↓Kv1.6, ↓Kv4.2, ↓KvLQT1, ↓SK1, ↓Kvβ1, ↓MiRP3, ↓MiRP4, ↓Kir3.1, ↓Kir3.4, ↓Kir6.1, ↓Kir6.2, Ca^2+^ channel and Ca^2+^-handling protein transcripts, including ↓Cav1.3,↓Cavβ3, ↓Cav-α2δ1, ↓NCX1, ↓Calm3, and ↓RIP3-2 | Mice | Hao *et al*. [28] |
| Ion channel remodelling measured using patch-clamp recordings from isolated SAN | ↓Conductance densities *I*_Ca,L,_ *I*_Ca,T_  Negative shift of activation midpoint for *I*_f_ | Mice | Larson *et al.* [27] |
| Ion channel remodelling detected through mRNA and protein expression measurement | expression of HCN channel subunits  (↓ HCN1) | Canine | Du *et al*. [25] |
| Ion channel remodelling using western blot. | ↑Cav1.2 protein expression | Guinea-pig | Jones *et al*. [19] |
|  | ↑Cav1.2, ↓RyR2, ↑SERCA2a, ↓NCX, ↓HCN4, ↑KvLQT1↓, ERG, ↓Kv4.2, ↑Cav3.1 , ↑Na^+^/K^+^ | Rats | Tellez *et al*.[31] |
| Ion channel remodelling measured using relative change in protein expression density | ↓Cav1.2, ↓HCN2, ↓HCN4  ↓RyR2, ↓SERCA2a, ↑NCX | Rats | Hatch. [32] |
| Ion channel remodelling through recording relative change in protein expression | expression of HCN channel subunits  (↓HCN2, ↓HCN4) | Rats | Huang *et al*.[33] |

**Table 2**. Relative changes in mRNA, protein expression levels and current density/channel conductance of ion channels and intracellular Ca^2+^ handling between adult and older adult rat SAN. Data are from studies on rat central SAN cells by Tellez *et al*. [31] (Ageing Study-1); Hatch*.* [32], Huang *et al.* [33] (Ageing Study-2). Arrows indicate up- or down-regulation of the underlying channels, as indicated by changes in mRNA, protein expression levels or measured channel current densities

| **Ageing Study -1** | | | **Ageing Study -2** | | | |
| --- | --- | --- | --- | --- | --- | --- |
| Isoform | \|Relative change in mRNA *(older adult/ adult) ×100* | Reference | Isoform | Relative change in protein expression *(older adult - adult)/ adult * 100.* | Relative change in protein expression density *(older adult - adult)/ adult * 100.* | Reference |
| Cav1.2 | ↑25% |  | \| Cav1.2 \| ↓40% \| ↓50% \| \| --- \| --- \| --- \| | \| ↓40% \| ↓50% \| \| --- \| --- \| | \| ↓50% \| ↓50% \| \| --- \| --- \| | Hatch.[32] |
| RyR2 | ↓80% |  | RyR2 | ↓67% | ↓24% |  |
| SERCA2a | ↑15% |  | SERCA2a | ↓83%  '7 | ↓29% |  |
| NCX | ↓6% |  | NCX | ↑72% | ↑42% |  |
| SERCA2a | ↑15% |  | HCN4 | ↓49% |  | Huang *et al*.[33] |
| NCX | ↓6% |  | HCN2 | ↓30% |  |  |
| HCN4 | ↓16% |  |  | | | |
| KvLQT1 | ↑60% |  |  |  |  |  |
| ERG | ↓8% |  |  |  |  |  |
| Na^+^/K^+^ Na+/K+ pump | ↑50% |  |  |  |  |  |

**Table 3**. Characteristics of action potentials and heart rates in adult and older adult conditions based on experimental data of Ageing Study-1 and Ageing Study-2, which were incorporated into the Tao *et al.*[35]model. Simulation data were compared with the experimental data of Tellez *et al.* [31] for Ageing Study-1 and Hatch. [32] for Ageing Study-2. Data with error bars were from the experimental studies of Tellez *et al.* [31] and Hatch*.* [32]

|  | **Adult**  **Simulation**  Tao *et al.*[35] | **Adult**  **Experiment**  Shinagawa *et al.*[40] | **Ageing study -1**  **Simulation** | **Ageing study -1**  **Experiment**  Tellez *et al.* [31] | **Ageing study -2**  **Simulation** | **Ageing study -2**  **Experiment**  ^+^Hatch*.*[32]  ^++^Huang *et al.* [33] |
| --- | --- | --- | --- | --- | --- | --- |
| dV/dt_max_(V/s) | 6.50 | 7.00 ± 1.0 | 10.85 | 11.00 ±1.0 | 2.31 | **-** |
| MDP (mV) | -54.01 | -56.02 ± 2.0 | -56.84 | **-** | -45.70 | - |
| PA (mV) | 20.00 | **-** | 24.58 | **-** | 4.16 | **-** |
| APD_50_ (ms) | 77.06 | 78.00 ± 1.0 | 88.00 | 90.14 ± 2.2 | 64.00 | **-** |
| CL (ms) | 230.00 | 232.23 ± 10.0 | 310.44 (↑26%) | 266.00 ± 10.0 (↑30%) | 265.22 (↑15.2%) | ^+^314.00± 12.0(↑40%)  ^++^235 ±4.0 (↑21%) |
| HR (bpm) | 260.00 | 258.62 ± 17.0 | 210.12 (↓19%) | 225.17 ± 14 (↓18%) | 226.45 (↓12.9%) | ^+^191.00±0.2(↓30.0 %)  ^++^255 ±5.0 (↓17.7%) |


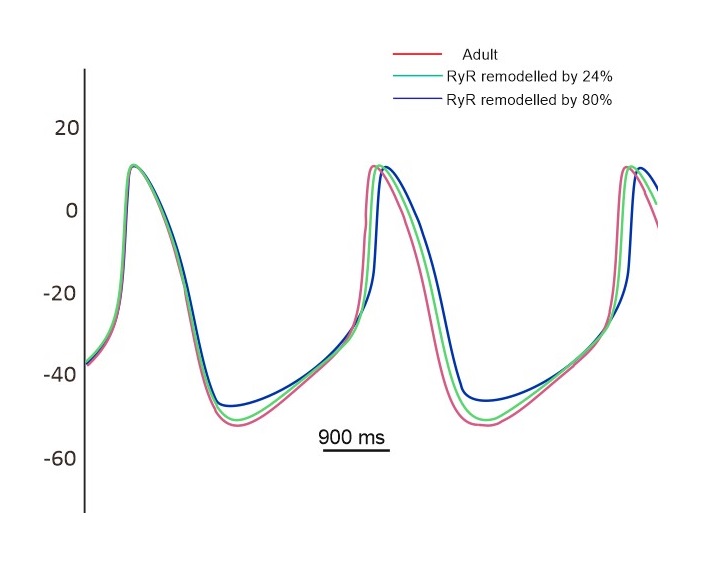


**FigureS1.** Simulated role of reduced Ca^2+^ release from the SR in ageing bradycardia using Maltsev *et al*.[46] model. Changes in other ion-channel currents as reported by Tellez *et al*. [31] in Ageing Study-1 (right panels) and by Hatch. [32] and Huang *et al*. [33] in Ageing Study-2 (left panels) were omitted.
